# Accuracy of retrospective pain measurement in patients with chronic pain

**DOI:** 10.3892/mi.2023.95

**Published:** 2023-07-04

**Authors:** Ingo Haase

**Affiliations:** Department of Research, Development and Quality Assurance, Clinic Group Enzensberg, D-87629 Hopfen am See, Germany

**Keywords:** chronic pain, pain intensity, pain measurement, recall, patient assessments, patient reported outcome

## Abstract

The use of pain scales that refer to a past time period is thereby based on the assumption that patients accurately remember their ‘average’, ‘greatest’ and ‘least’ pain. The present study addresses the validity of numerical pain rating scales that refer to a past period of time (herein, the past 7 days). Routine data from 94 patients with chronic pain were retrospectively analysed. Pain questionnaire data on the greatest, least and average pain during the past week and on current pain were compared with the mean value of entries in a pain diary from the corresponding period. The retrospectively assessed average, greatest and least pain values were consistently slightly higher than the corresponding values of daily current pain measured for the studied collective of chronic pain patients. Current pain (at the time of answering the questionnaire) better represents daily currently measured pain [intraclass correlation (ICC)=0.885] than retrospective individual measurements. The greatest correlation with averaged diary data was shown by the combination of questionnaire data on average, least and current pain (ICC=0.911). The high correlations between the questionnaire and diary data support the validity of retrospective pain surveys. However, the current status influences recall. Thus, composite retrospective pain data improve with the addition of current pain.

## Introduction

Self-assessments of pain intensity using visual analogue scales (VAS), numeric rating scales (NRS), or verbal rating scales (VRS) are widely used in pain management and research. The use of pain scales that refer to a past time period (often the last 7 days) is thereby based on the assumption that patients accurately remember their ‘average’, ‘greatest’ and ‘least’ pain. However, the validity of such memory ratings do not provide a clear picture.

It has been found that retrospective single-recall ratings of average pain over the 1-week period are valid and feasible in the studies by Bolton (1) for back pain, Bolton *et al* (2) for neck pain, Jamison *et al* (3) for low back pain, Perrot *et al* (4) for coxarthrosis and gonarthrosis, and Jensen *et al* (5) for chronic pain.

By contrast, the results of the study by Giske *et al* (6) for musculoskeletal pain and Stone *et al* (7) for chronic pain raise doubts about the validity of pain recalled after 1 week. According to the study by Broderick *et al* (8), the pain recall of patients with rheumatoid arthritis after 1 week is worse than that after 1 day, after 3 days and after 28 days.

Against this background, the aim of the present study was to compare daily current and weekly recalled pain ratings on NRS in patients with chronic pain at different pain locations. In detail, the present study aims to address the following questions: i) Whether pain is recalled at similar levels to diary ratings; ii) which combinations of the individual pain ratings correspond best with the average of the daily current measurements using the pain diary; and iii) possible factors that influence the recollection of pain.

## Patients and methods

### Patient data

The present retrospective study is a secondary analysis of anonymized routine data from an interdisciplinary pain centre in Germany. The data analysed herein were collected within the time frame between August, 2013 to December, 2014. The study was conducted in accordance with the requirements of the Declaration of Helsinki as far as they apply to retrospective studies. No study-related interventions were performed on humans or animals. For the present retrospective study, only data that are already implemented in routine care were used in an anonymized form (no code list available, no personal reference can be established). The patients provided written consent. The data were treated according to the German and the Bavarian legislative rules for data protection. Routine data collection accompanies therapeutic action at the Specialized Clinic Enzensberg (Füssen, Germany) as a matter of quality assurance. This is in accordance with the Ethics Committee of the Bavarian Medical Association.

The included patients with chronic pain completed both pain questionnaires (including at discharge) and pain diaries (daily) with NRS values for pain measurement. Only cases with complete pain ratings in the German Pain Questionnaire (DSF) and no more than seven missing items (25%) in the pain diary (in the week before discharge) were included in the analysis. Individual missing values were not replaced. The realized case number of n=94 is sufficient to statistically support a mean association at a power >90% with α=0.05.

The average currently experienced pain intensity according to the diary was calculated as the mean of up to four statements per day over a course of 7 days (arithmetic mean). The retrospective assessment of the greatest, least and average pain during the last week before discharge, as well as the current pain were collected using a questionnaire (DSF).

### Data and statistical analyses

The recall ratings were examined in two ways: First, it was examined whether there were level differences between the retrospective ratings and the average diary information, and second, the association between the retrospective and the daily current information on pain intensity was analysed. The underlying numeric rating scales are considered to be quasi interval scaled. Statistical analyses for this included descriptive statistics (mean and standard deviation), the Wilcoxon signed-rank test for two related paired samples, correlation analyses (intraclass correlation), and multiple linear and logistic regression analyses. Multiple linear regression analyses were performed with the arithmetic mean of daily current pain diary entries as the dependent variable. The dichotomized difference between the average current pain intensity (diary) and the recalled average pain intensity (questionnaire) was defined as the dependent variable for the logistic regression. For differences of less than one point, recollection was considered equivalent to average current diary entries. Only variables exhibiting significant differences between groups in the pre-test (P≤0.05 in Pearson's Chi-squared test for categorical variables and the Kolmogorov-Smirnov-Z-Test for quantitative variables) were included in the logistic regression. All analyses were performed using the SPSS statistical software package, version 21 (IBM Corp.).

## Results

The investigated collective comprised 94 patients with chronic pain of the chronification stages II and III according to the Mainz Pain Staging System (9), who were treated as inpatients in an interdisciplinary pain centre in Germany. The most frequent pain localizations according to the patients were back pain (including neck pain) in 45% of the patients, whole body pain in 17%, joint pain in 14% and headache in 7%. The other key characteristics of the patient sample in the present study are presented in Table I.

Comparison of pain levels: The recalled pain (according to the answers in the pain questionnaire) is consistently higher than the corresponding pain measured on a daily basis (mean of pain diary data). This is true for the recollection of the average pain, as well as for the greatest pain and the least pain of the last 7 days, in comparison with the mean of the diary entries, respectively the minimum and maximum of the daily measurements in the last 7 days (Table II). Thereby, the differences of the recollection from the documented current pain were significant for the greatest pain and the average pain (Wilcoxon test, Z=-4.027 and Z=-4.134, respectively; P-values <0.001), whereas the small difference for the least pain was not (P=0.057).

Among the four pain statements in the questionnaire, momentary pain (while filling in the questionnaire) was closest to the averaged diary statements, followed by recalled average pain in the last 7 days. The values for current pain at the time of the retrospective assessment were almost identical to the mean value of the diary statements (Table II).

As regards the combination of pain statements in the questionnaire, all variants and combinations of recalled pain correlated with the mean of the daily current measurements, as was expected (Table III). The intraclass correlation (ICC) of the retrospective single measurements and the weekly average varied from 0.181 (greatest pain) to 0.779 (average pain). For the possible combinations of the retrospective individual intercepts, the combination of average and least pain exhibited the highest ICC of 0.876, and the combination of average and greatest pain exhibited the smallest value (ICC=0.536).

The addition of the current pain strengthened the correlation. As a single rating, the current pain statement outperforms all individual recall ratings with an ICC of 0.885. The composite recall ratings each improve with the addition of current pain (Table III). The highest ICC value was obtained by the combination of average, least and current pain (ICC=0.911) (Table III).

The influence of momentary pain was confirmed by multiple linear regression analysis. It was proven to be the most important predictor of the averaged diary data on current pain (β=0.441, P<0.001). Other predictors included in the model were least pain in the last 7 days (β=0.383, P<0.001) and average pain in the last 7 days (β=0.155, P=0.043) (Table IV). In contrast, remembered greatest pain did not play a role.

A deviation defined as striking between the recalled average pain and the mean from the diary data of the corresponding period of at least one point was shown in 35 cases (37%).

Since bivariate pre-tests did not find an effect of different variables - sex, age, body mass index, schooling, employment status, pension application, main diagnosis, number of secondary diagnoses, pain duration, baseline pain, tolerable pain, perceived impact of pain, impairment due to pain (pain disability index), physical and psychological quality of life (SF-36 summary scores), depressive symptomatology (Center for Epidemiologic Studies Depression Scale), length of stay-regarding recall accuracy (all P-values >0.15), the originally planned logistic regression analysis was omitted.

## Discussion

Pain intensity is a main criterion for assessing the effectiveness of pain therapy (4). Determining this requires dealing with methodological questions of pain measurement, particularly since the variable pain intensity is an often discussed and criticised variable. Due to the lack of objective measurement options, there is still no way around interviewing the affected patients.

Traditional pain measurement requires patients to recall their pain intensity over a past time period of days or weeks. However, the recall of pain experience is variable (10) and complex (11). Retrospective assessments may therefore be biased mostly toward the overestimation of pain intensity (4). The findings in the study by Berger *et al* (12) suggest that a circuit localized in the hippocampus and personality traits associated with processes in the reward system are significantly responsible for chronic pain patients retrospectively overestimating their daily pain sensations.

The data from the present study also demonstrated that the average, greatest and least pain assessed retrospectively (with respect to the past 7 days) were consistently higher than the corresponding value of the daily current pain measured. Momentary pain (at the time of answering the questionnaire) better represents daily currently measured pain than retrospective single measurements. Thus, the current status apparently influences recall and, incidentally, the perception of change (13). That recall of pain being influenced by current pain has already been shown for patients with chronic low back pain (14) and rheumatism (15), as well as hip and knee osteoarthritis (4).

In addition to the importance of current pain for the assessment of pain in a past time period, the lack of validity of the recall of the greatest pain is noticeable. It exhibits a significantly lower correlation with the averaged diary entries than the memories of the average and least pain, and compared with the current pain perception. Accordingly, the validity of the composite pain intensity ratings suffers when the information on the greatest pain is included. By contrast, the addition of the assessment of current pain improves the respective composite models. The query of the greatest pain thus appears to be dispensable for the majority of pain disorders - at least as regards the period of the last 7 days examined herein. The query of the greatest pain could be helpful; however, averaging is not very meaningful in the case of long pain-free phases, such as in trigeminal neuralgia or cluster headache.

The arithmetic mean of the questionnaire data on average, least and current pain best represents the current average pain intensity recorded via a pain diary with excellent agreement (ICC=0.911). However, this does not exclude the use of single retrospective ratings. Thus, the individual assessment of average pain with an ICC of 0.779 also shows a validity that can be considered good (16). Moreover, it is more feasible and economical for measuring a patient's pain intensity over a period of 1 week due to its unidimensionality (17), and is recommended for patients with non-specific low back pain. An expert group on the standardization of outcome reporting in clinical trials of non-specific low back pain achieved a high consensus (96%) in a two-stage Delphi process for questioning average pain intensity in the past 7 days using NRS (18).

However, when the greatest possible validity is important, such as in clinical trials, composite pain intensity scales should be used, which are superior to single scales (19).

That the recollection of pain is independent of various sociodemographic, medical and psychological variables from the available routine, is indicative of the generality of the present findings in the framework studied. However, it cannot be excluded that other variables not present herein influence recall. Elsewhere, the most severe pain experienced and current mood (12) in chronic back pain patients, as well as catastrophizing pain and neuroticism (20), have been identified as factors biasing recall of acute pain experience in young healthy adults. The use of novel digital technologies may be able to reduce recall bias and improve the real-time detection of acute and chronic symptoms (21).

The present study has some limitations which should be mentioned. The present analysis refers to patients with chronic pain of higher chronicity who report pain intensity in the past 7 days on NRS. The results are not generalizable to other patient groups and outcome periods. In addition, the use of other pain scales (VAS and VRS) may lead to differential results. Furthermore, it should be mentioned as a limitation that not all pain diaries were fully completed; 19% of the cases studied had more than two and thus more than 10% missing information. Another limitation is the complexity of the pain experience. If, as the results of Broderick *et al* (11) suggest, patients included impairments in the physical and social domains in the information about pain intensity in the past week, then the validity of the retrospective pain query as a measure of pure pain intensity would not be given (22). The pain diary entries are likely to be less affected by this, as they are point measures in which impairments due to the pain experienced are less of an issue. The high correlations shown in the present study thus argue for the sufficient validity of retrospective pain surveys, ideally as a combination of the average, least and current pain data.

## Figures and Tables

**Table I:** Characteristics of the present study sample.

Characteristic	Included patients (n=94)
Sex, n (%)	
Females	72 (77%)
Males	22 (23%)
Age (years)	53.0 (range, 28-81; SD=10.5)
School-leaving qualification, n (%)	
No degree	7 (7%)
Secondary school	43 (46%)
Middle school	32 (34%)
High school/University degree	12 (13%)
Employed (full- or part-time), n (%)	51 (54%)
Duration of pain (years)	9.5 (range, 0.3-45; SD=10.5)
Average pain intensity (baseline)	6.9 (range, 3-10; SD=1.8)
Body mass index	26.7 (range, 17.7-41.9; SD=5.2)
Length of stay (days)	27.6 (range, 15-42; SD=3.7)

SD, standard deviation.

**Table II:** Comparison of questionnaire data and averaged diary data on pain intensity.

Pain intensity	Questionnaire mean (SD)	Pain diary mean (SD)	Significance P-value^a^
Least pain according to questionnaire response; minimum diary entry	3.4 (2.2)	3.2 (2.3)	0.057
Greatest pain according to questionnaire response; maximum diary entry	7.1 (1.9)	6.4 (2.3)	<0.001
Average pain according to questionnaire response; average diary data	5.3 (1.9)	5.3 (1.9)	<0.001
Current pain according to questionnaire (when filling in)	4.8 (2.3)	Not applicable	

^a^Data were analysed using the Wilcoxon signed-rank test. SD, standard deviation.

**Table III:** Correlations of various retrospective pain ratings (questionnaire) with current pain ratings (diary data).

Retrospective pain ratings individually and combined (arithmetic mean)	ICC
Average/least/current pain	0.911
Current pain^a^	0.885
Average/current pain	0.882
Least/current pain	0.881
Average/least pain	0.876
Most/least/current pain	0.870
Average/most/least/current pain	0.866
Average/most/least pain	0.816
Most/least pain	0.800
Average pain^a^	0.779
Average/most/current pain	0.735
Least pain^a^	0.721
Most/current pain	0.668
Average/most pain	0.536
Most pain^a^	0.181

^a^Indicates all single ratings. ICC, intraclass correlation coefficient.

**Table IV:** Results of regression analysis.

Predictors	β	Corrected R^2^ value
Current pain (questionnaire)	0.441^b^	0.782^b^
Least pain (questionnaire)	0.383^b^	0.839^b^
Average pain (questionnaire)	0.155^a^	0.845^b^

^a^P<0.05 and

^b^P<0.001, indicate significant differences.

## Data Availability

The datasets used and/or analysed during the current study are available from the corresponding author on reasonable request.
